# Difference in accuracy of lung sliding identification between the right and left hemithorax

**DOI:** 10.1186/cc10694

**Published:** 2012-03-20

**Authors:** R Daoust, E Piette, J Lambert, A Denault

**Affiliations:** 1 Hôpital du Sacré-Coeur de Montréal, Canada; 2 Université de Montréal, Canada; 3 Institut de Cardiologie de Montréal, Canada

## Introduction

The field of lung ultrasound (US) in critical care is in rapid expansion. Lung sliding (LS) identification has been used in emergency medicine (EM) to diagnose pneumothorax as well as to evaluate the adequacy of endotracheal intubation. Presence of the Lung Pulse artefact (back and forth pleural motion induced by the heartbeat) as well as the underlying heart may affect correct identification of LS in the left hemithorax, but this has never been studied. Our main objective was to evaluate the rate of correct identification (accuracy) of the presence or absence of LS in the right and left hemithorax.

## Methods

A total of 280 short lung US sequences (one respiratory cycle), recorded in the operating room, of presence and absence of LS in intubated patients were randomly presented to two groups of physicians (in total: two medical students, 42 EM residents and 31 EM attendings). Sequences were divided equally between the right and left hemithorax. Each participant's knowledge of the Lung Pulse artefact was noted. Only the second group was instructed not to answer in case of uncertainty. A Kolmogorov-Smirnov test showed the rate of correct LS identification did not follow a normal distribution. Median rates are reported with interquartile range (IQR) and compared using a Mann-Whitney test.

## Results

Knowledge of Lung Pulse was higher in the second group (55% vs. 21%, *P *< 0.05). Globally, median accuracy of identification of LS presence or absence was 74.0% (IQR: 48.0 to 90.0) in the first group and 83.7% (IQR: 53.3 to 96.2) in the second (*P *= 0.006). For the first group, median accuracy was 80.0% (IQR: 57.0 to 95.0) in the right hemithorax and 67.0% (IQR: 43.0 to 83.0) in the left (*P *< 0.001). For the second group, median accuracy was 88.7% (IQR: 63.1 to 96.9) in the right hemithorax and 76.3% (IQR: 42.9 to 90.9) in the left (*P *< 0.001).

## Conclusion

Accuracy of identification of LS presence or absence is higher in the right hemithorax. Our study is the first to report this finding. Presence of the Lung Pulse artefact, as well as the underlying heart, probably explains the worse accuracy found in the left hemithorax. Caution should be taken in using LS identification as a diagnostic tool in the left hemithorax and knowledge of the Lung Pulse artefact should be emphasized in chest US curriculum.

